# Effect of Meditation, Mindfulness-Based Stress Reduction, and Relaxation Techniques as Mind-Body Medicine Practices to Reduce Blood Pressure in Cardiac Patients: A Systematic Review and Meta-Analysis

**DOI:** 10.7759/cureus.58434

**Published:** 2024-04-17

**Authors:** Dapkupar Wankhar, Archana Prabu Kumar, Venugopal Vijayakumar, Velan A, Arthi Balakrishnan, Poornima Ravi, Bhandari Rudra, Maheshkumar K

**Affiliations:** 1 Physiology, People’s College of Medical Sciences and Research Centre, Bhanpur, IND; 2 College of Medicine and Medical Science, Arabian Gulf University, Manama, BHR; 3 Yoga, Government Yoga and Naturopathy Medical College and Hospital, Chennai, IND; 4 Yoga, International Institute of Yoga and Naturopathy Medical Sciences, Chengalpattu, IND; 5 Naturopathy, International Institute of Yoga and Naturopathy Medical Sciences, Chennai, IND; 6 Science of Yoga, University of Patanjali, Haridwar, IND; 7 Physiology and Biochemistry, Government Yoga and Naturopathy Medical College and Hospital, Chennai, IND

**Keywords:** pranayama, systematic review and meta analysis, cardio vascular disease, integrative approach, cam therapy, hypertension, blood pressure, relaxation, yoga, mind body medicine

## Abstract

Elevated blood pressure is one of the major risk factors for cardiovascular diseases. Available evidence on mind-body medicine (MBM) techniques on blood pressure is inconclusive and provides conflicting results. The objective of the current systematic review and meta-analysis is to evaluate the effect of MBM techniques on blood pressure in patients with cardiovascular disease. Randomized control trials (RCTs) done between the years 2000 and 2020 on cardiovascular disease, using MBM techniques such as meditation, mindfulness-based stress reduction and relaxation techniques were searched through electronic databases such as PubMed, Cumulative Index to Nursing & Allied Health (CINAHL), EMBASE and Cochrane Library. Three authors independently performed article selection, data extraction and validation. Meta-analysis was performed using a random effect model and standardized mean difference (SMD) with 95% confidence interval (CI) estimated for the effect size. Fifteen RCTs with 927 patients were included in the meta-analysis. Heterogeneity among the studies was very high for all analyses (I^2^>94%). For studies comparing systolic blood pressure, MBM interventions show a significant (p=0.01) effect when compared to conventional treatment, an overall estimated effect size of SMD - 0.78 (95% CI: -1.36, -0.20). For studies comparing the diastolic blood pressure, MBM intervention did not show any significant effect when compared to the conventional treatment, an overall effect size of SMD -0.26 (95% CI: -0.91, 0.39). The findings of the meta-analysis suggest that MBM interventions may improve systolic blood pressure alone in patients with cardiac diseases. With high heterogeneity and low quality of the included studies, more robust evidence is required before suggesting MBM as an effective treatment modality for reducing blood pressure in cardiovascular diseases.

## Introduction and background

Cardiovascular disease (CVD) is the world's leading cause of morbidity and mortality. It refers to a wide range of illnesses that affect the heart and blood vessels [1]. Elevated blood pressure is the major risk factor that leads to cardiovascular disease onset and its consequences [2]. All cardiovascular diseases, particularly stroke, heart attack, heart failure and myocardial infarction are linked with elevated blood pressure. Hypertension (HTN) can lead to end organ failure which is clinically represented as transient ischemic attack, cerebral infarction, intracerebral or subarachnoid bleed, acute coronary syndrome, acute kidney injury and haemorrhage [3]. Anxiety, stress, depression and anger influence onset and severity of hypertension and cardiovascular disorder [4]. People with stress, anxiety, and depression are more prone to develop HTN. Psychological stressors affect autonomic and neuroendocrine functions, altering levels of serum cortisol and cytokines, and producing chronic inflammation in blood vessels [5]. Evidence correlating mental stress to endothelial dysfunction shows increased peripheral microvascular tone and vasoconstriction of the coronary artery [6]. Many challenges are faced by healthcare practitioners in attaining blood pressure control, including patient difficulty in consuming various drugs to meet blood pressure standards [7]. Mind-body medicine (MBM) therapies involve interaction between brain, mind and body. Meditation and other mind-body practices such as mindfulness and yoga are gaining increasing significance, with over 14% of the adults in U.S. having undergone one of these interventions [8]. MBM is documented to be beneficial in the management of blood pressure and cardiovascular diseases [9,10]. MBM practices produce a cascade of physiological responses like reduced oxygen consumption and lowered blood pressure and heart rate [11]. Previous studies with MBM interventions showed beneficial effects in migraine, psychological disorders, chronic pain management and neurological disorders, but there is a lack of evidence to show the efficacy of MBM on patients with cardiovascular diseases [11-13]. Therefore, the objective of this meta-analysis is to investigate the effects of MBM interventions on patients with cardiovascular diseases like coronary artery disease, stable angina, acute myocardial infarction, arterial hypertension and chronic heart failure for blood pressure control. To validate the recent updates we restricted the search between the years 2000 and 2020.

## Review

Methodology

Search Strategy

This systematic review and meta-analysis was designed, performed and reported in accordance with the Preferred Reporting Items for Systematic Review and Meta-Analysis (PRISMA) guidelines [14]. A comprehensive literature search was done using electronic databases such as PubMed, Scopus, Cochrane Library, Prospero, and Embase. The randomized control trials focusing on MBM interventions that were published between the years 2000 and 2020 were included. We used a combination of search terms like “Mind-Body Medicine”, OR “Meditation”, OR “Mindfulness based stress reduction”, OR “Relaxation Techniques”, OR “Breathing technique”, AND “Hypertension”, OR “Blood Pressure”, OR “Coronary Artery Disease” OR “Cardiovascular diseases” and Medical Subject Headings MesH term ("Mind-Body Therapies"[Mesh]) AND "Cardiovascular Diseases"[Mesh]. Three authors (AV, PR and AB) independently evaluated title and abstract to discover papers that met the inclusion and exclusion criteria and then access the full text of the articles that matches the requirements. Any disagreements about study selection or possible rearrangements were resolved by discussion between three reviewers, including the corresponding author (MK), in order to avoid inaccuracies, if any.

Selection of Studies

Inclusion criteria: All the studies which had met the following PICO criteria were included: (I) Participants (P): Patients diagnosed with one of the cardiovascular diseases such as hypertension, heart failure, myocardial infarction, and other coronary artery diseases. (II) Intervention (I): Mindfulness techniques, relaxation techniques, meditation techniques, and mindfulness-based stress reduction. (III) Comparator group (C): Health education, social programs, a waiting list, and routine care with or without drugs are provided to the control group. (IV) Outcomes (O): Systolic blood pressure and diastolic blood pressure were measured using the standard method.

Exclusion criteria: Patients who did not have cardiovascular disease were not included in the study. Additionally, studies with non-randomized designs, articles in languages other than English, and studies published before 2000 were excluded. Furthermore, studies that employed interventions or therapies other than MBM were not considered for inclusion.

Data Extraction and Reliability

Two authors independently extracted data from studies and assessed them using Excel (Microsoft, Redmond, WA, USA). The extracted data included information such as author, year, country, age, sample size, study design, details of interventions for the experimental group, details of interventions for the control group, and outcome parameters. Only studies comparing mind-body practices with a control group containing more than one practice or control arm were included in the analysis.

Assessment of Risk of Bias

Three investigators (AV, PR, and AB) evaluated the methodological quality of the included studies following the guidelines outlined in the Cochrane Handbook for assessing the risk of bias in randomized trials. Key domains assessed included random sequence generation, allocation concealment, blinding of participants and personnel, blinding of outcome assessment, presence of other sources of bias, completeness of outcome data, and selective outcome reporting. These domains were evaluated across all included studies to ensure a comprehensive assessment of methodological quality [15].

Statistical Analysis

The mean differences in systolic blood pressure (SBP) and diastolic blood pressure (DBP) between experimental and control groups were computed. Standardized mean differences (SMD) with 95% confidence intervals (CI) were calculated to determine the effect size. Heterogeneity was assessed using the I^2^ statistics and Cochran Q test, and a random-effect model was employed to improve result accuracy. Egger tests were conducted to identify potential publication bias among the included studies, with a significance level set at 0.05. Additionally, potential publication bias was assessed using a funnel plot, where an asymmetric inverted funnel-shaped scatter plot of treatment effects against study size could indicate publication bias or systematic heterogeneity. The analysis was conducted using the metafor package in R statistical software version 4.2.1 (R Foundation for Statistical Computing, Vienna, Austria) [16].

Results

Literature Selection

**Figure 1:**
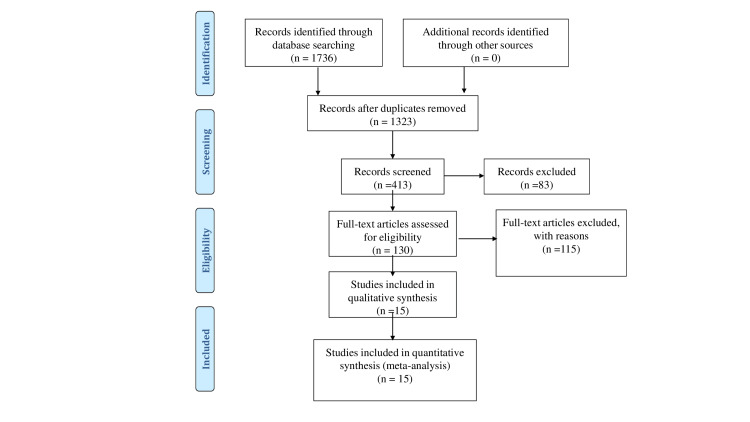
Preferred Reporting Items for Systematic Reviews and Meta-Analyses (PRISMA) diagram illustrating the search strategy for the review

A total of 1736 articles were initially identified through the literature search. Following the screening of titles and abstracts by three independent authors, 1606 articles were excluded. Subsequently, the full text of 130 potential articles was carefully reviewed, resulting in the inclusion of 15 articles that met the predefined inclusion criteria for the meta-analysis (Figure 1).

Literature Characteristics

Table 1 shows the literature characteristics of the included studies. Fifteen studies are included for the current meta-analysis and all the trials were done between the years 2000 to 2020, out of which five studies were done in the USA [17-20], two studies in Iran [21,22], and the rest in Brazil [23], Los Angeles [24], Portugal [25], Iowa [26], India [27], Spain [28], Italy [29], and Canada [30]. The age of the participants ranges from 30 to 80 years. All the studies included both male and female participants except one study which included only male participants [27]. The minimum sample size in both the study and control group was 15 each, while the maximum sample size in the study group was 99 and the control group was 102. A total of seven studies recruited hypertensive patients [17-20,22,28,29], five studies included patients with coronary artery diseases [21,26,27], two studies were done on patients with heart failure [23,29] and one study included both patients with coronary artery disease and heart failure [31]. Around seven studies had meditation as their intervention [17,19,21,24,26,28,31], five studies gave mindfulness-based stress reduction (MBSR) [18,22,24,25,31], and two studies gave breathing technique [17,29] as an intervention. Control group was given usual care, health education, rehabilitation therapy and psychological therapy. Minimum duration of intervention was four weeks and maximum duration was 16 weeks. Most commonly used parameters for assessment in all the studies were systolic and diastolic blood pressure.

**Table 1:** Characteristics of the studies included in the meta-analysis RCT- Randomized controlled study; CHF- Chronic heart failure; HTN- Hypertension; CAD- Coronary artery disease; MI- Myocardial infarction; CHD- Congestive heart disease

Author	Country	Age	Sex	Sample Size	Study Design	Intervention for experimental group	Intervention for control group	Intervention details	Condition
Anderson DE et al., 2010 [17]	USA	53.4 (2.8 )	Both	E-20, C-20	RCT	Device guided breathing	Natural breathing	15 minutes /day/ 4 weeks	HTN
Palta P et al., 2012 [18]	USA	72.3 (4.4 )	Both	E-12, C-8	RCT	Mindfulness based stress reduction	Social support	90 minutes /week/ 8 weeks	HTN
Duraimani S et al., 2015 [19]	USA	60.4 ( 11.9 )	Both	E-24, C-24	RCT	Transcendental meditation	Health education	20 minutes / 16 weeks	HTN
Schneider RH et al., 2019 [20]	USA	53.3 (9.4 )	Both sex	E-41, C-44	RCT	Transcental meditation	Health education	20 minutes twice a day/3 months	HTN
Delui MH et al., 2013 [21]	Iran	40 -65	Both	E-15, C-15	RCT	Mindfulness Meditation	Usual care	20- 25 minutes/ 10 sessions	CAD
Momeni J et al., 2016 [22]	Iran	47 ( 7 )	Both	E-30, C-30	RCT	Mindfulness based stress reduction	Psychological therapy	2.5 hour/week/8 weeks	HTN
Curiati JA et al., 2005 [23]	Brazil	74.8 (6.7)	Both	E-8, C-7	RCT	Meditation techniques	Weekly meeting	30 minutes/ 12 weeks	CHF
Maura Paul-Labrador M et al., 2006 [24]	Los Angeles	67.7 (9.0)	Both	E-52, C-51	RCT	Transcendental Meditation	Health education	1.5hours /16 weeks	CHD
Neves A et al., 2009 [25]	Portugal	59.5. ( 10.8 )	Both	E-40, C-41	RCT	Relaxation & imaginary relaxation techniques	Cardiac rehabilitation	1 hour / 12 weeks	MI
Schneider RH et al., 2012 [26]	Iowa	59.9 (10.7 )	Both	E-99, C-102	RCT	Transcendental meditation	Health education	20 minutes /3 months	CAD
Marquez PH et al., 2019 [28]	Spain	56.5( 7.7 )	Both sex	E-24, C-18	RCT	Mindfulness Meditation	Health education	2 hours /week/8 weeks	HTN
Drozdz T et al., 2015 [29]	Italy	65.5 ( 11.6 )	Both	E-20, C-20	RCT	Slow breathing training	Usual care	15 minutes /session/ 12 weeks	CHF
Blom K et al., 2014 [30]	Canada	57 ( 12 )	Both	E-46, C-41	RCT	Mindfulness based stress reduction & Yoga	Waiting list	45 minutes /day/ 12 weeks	HTN
Nijjar PS et al., 2019 [31]	USA	58.6 (10.8 )	Both sex	E-30, C-15	RCT	Mindfulness based stress reduction	Usual care	2.5 hours/week/8 weeks	CAD CHF
Parswani MJ et al., 2013 [32]	India	30-65	Male	E-15, C-15	RCT	Mindfulness based stress reduction	Health education	1-1.5 hour/ 8 weeks	CAD

Risk of Bias Assessment

Table 2 presents an overview of the methodological quality assessment conducted for the included studies. With respect to random sequencing, all of them were rated low risk and 10 studies were rated low risk in general allocation concealment while in the rest (five studies) it’s unclear [19,21,23,25,28]. With regards to blinding of participants and personnel reporting, 12 studies rated low risk and one study had high risk [28]. Twelve studies were rated low risk in blinding of outcome assessment, while the rest (three studies) had high risk [23,28,29]. Other sources of bias, data-selective outcomes and incomplete outcomes are unreported.

**Table 2:** Risk of Bias assessment of included studies

AUTHOR	Random Sequence	Generation Allocation concealment	Blinding of participants and personnel reporting	Other sources of bias	Blinding of outcome assessment	Incomplete outcome	Data Selective outcome
Anderson DE et al., 2010 [17]	Low Risk	Low Risk	unclear	Low Risk	unclear	unclear	unclear
Palta P et al., 2012 [18]	Low Risk	Low Risk	Low Risk	Not clear	Low Risk	unclear	unclear
Duraimani S et al., 2015 [19]	Low Risk	Not clear	Low Risk	Not clear	Low Risk	unclear	unclear
Schneider RH et al., 2019 [20]	Low Risk	Low Risk	Low Risk	Low Risk	Low Risk	unclear	unclear
Delui MH et al., 2013 [21]	Low Risk	Not clear	Low Risk	Not clear	Low Risk	unclear	unclear
Momeni J et al., 2016 [22]	Low Risk	Low Risk	Low Risk	unclear	Low Risk	unclear	unclear
Curiati JA et al., 2005 [23]	Low Risk	Low Risk	Low Risk	High Risk	High Risk	unclear	High Risk
Maura Paul-Labrador M et al., 2006 [24]	Low Risk	Low Risk	Low Risk	Low Risk	Low Risk	unclear	Low Risk
Neves A et al., 2009 [25]	Low Risk	unclear	Low Risk	unclear	Low Risk	Low Risk	unclear
Schneider RH et al., 2012 [26]	Low Risk	Low Risk	Low Risk	Not clear	Low Risk	unclear	unclear
Marquez PH et al., 2019 [28]	Low Risk	Not clear	High Risk	unclear	High Risk	unclear	unclear
Drozdz T et al., 2015 [29]	Low Risk	Low Risk	Low Risk	High Risk	High Risk	Low Risk	High Risk
Blom K et al., 2014 [30]	Low Risk	Low Risk	Low Risk	High Risk	Low Risk	unclear	unclear
Nijjar PS et al., 2019 [31]	Low Risk	Low Risk	Low Risk	unclear	unclear	unclear	Unclear
Parswani MJ et al., 2013 [32]	Low Risk	Low Risk	unclear	Not clear	Low Risk	Low Risk	unclear

Meta-Analysis

**Figure 2:**
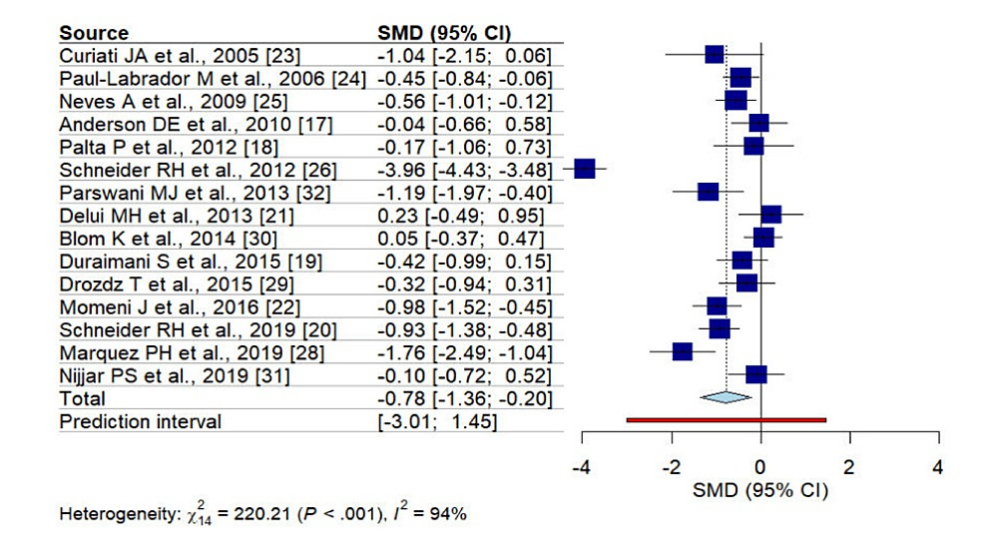
Forest plot for SBP within cardiovascular patients with MBM intervention SMD- standardized mean difference, SBP- systolic blood pressure, MBM- mind-body medicine

**Figure 3:**
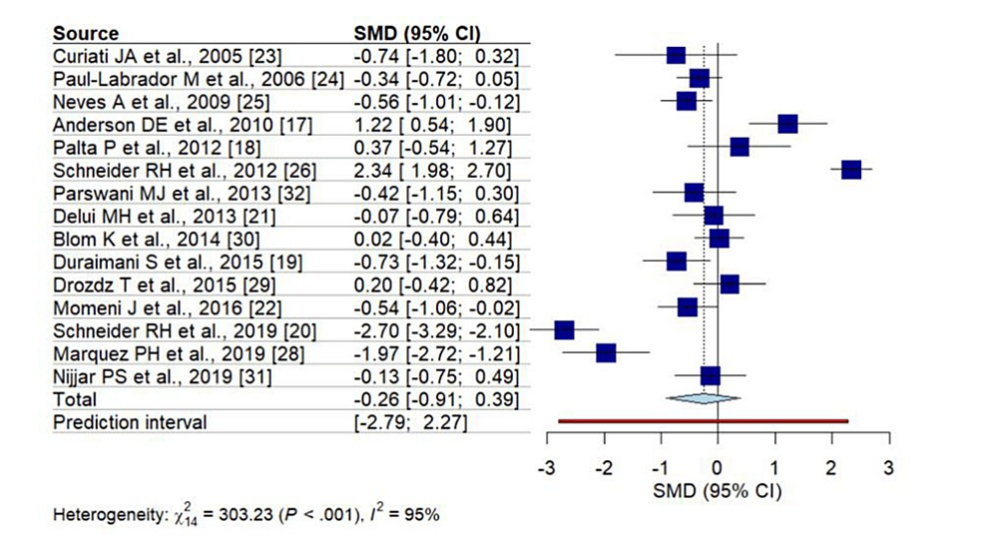
Forest plot for DBP in cardiovascular patients with MBM intervention SMD- standardized mean difference, DBP- diastolic blood pressure, MBM- mind-body medicine

Among the included studies, the combined results (Figure 2) revealed a significant effect on SBP (SMD: -0.78, 95% CI: -1.36, -0.20), with notable heterogeneity observed among the studies (I^2 ^94%, p < 0.001). Conversely, changes in DBP did not favor MBM intervention (Figure 3) (SMD: -0.26, 95% CI: -0.91, 0.39), and significant heterogeneity between studies was observed (I^2^ 95%, p < 0.001).

Subgroup Analysis

Subgroup analyses performed to examine the effect of MBM intervention on blood pressure changes showed systolic blood pressure to decrease effectively with frequency of less than eight weeks (SMD: -0.62, 95%CI=- -1.20, 0.05), with meditation (SMD: -1.76, 95%CI: -2.49, -1.04) and breathing technique (SMD: -0.78, 95CI: -1.36, -0.20) and compared with passive control group (SMD: -1.25, 95% CI: -2.28, -0.23). No such effects were noted for the MBM intervention on diastolic blood pressure changes (Table 3).

**Table 3:** Subgroup analysis by duration and frequency of mind-body medicine interventions CI- confidence interval, SBP- systolic blood pressure, DBP- diastolic blood pressure, I2- heterogenicity; P< 0.0.5 set as significant

Subgroups		SBP Mean difference in mmHg (95% CI)	P	I^2^	DBP Mean difference in mmHg (95% CI)	P	I^2^
Duration	>30 min	-0.56 (-1.02; -0.10)	0.03	72%	--0.26 (-0.90; 0.37)	0.71	82%
	<30 min	-1.09 (--2.66; 0.49)	0.42	97%	-0.27 (-2.00; 1.47)	0.52	98%
Frequency	> 8 weeks	-0.96 (-2.23; 0.32)	0.56	97%	0.05 (-0.96; 1.07)	0.23	96%
	< 8 weeks	-0.62 (-1.20, -0.05)	0.01	74%	-0.54 (-1.59; 0.51)	0.41	93%
Type of Intervention	Meditation	-1.76 (-2.49; -1.04)	0.02	96%	-0.59 (-2.08; 0.90)	0.65	98%
	Relaxation	-0.47 (-1.00; 0.05)	0.56	65%	-0.26 (-0.61; 0.09)	0.82	27%
	Breathing Techniques	-0.78 (-1.36; -0.20)	0.03	94%	-0.26 (-0.91; 0.39)	0.19	95%
Comparison Group	Passive control	-1.25 (-2.28; -0.23)	0.002	96%	-0.51 (-1.79; 0.76)	0.52	97%
	Active control	-0.55 (-1.68; 0.59)	0.81	60%	0.02 (-2.50; 2.53)	0.70	90%
	Usual care	-0.03 (-3.01; 1.45)	0.52	94%	0.01 (-0.20; 0.22)	0.35	0%

Publication Bias and Sensitivity Analysis

**Figure 4:**
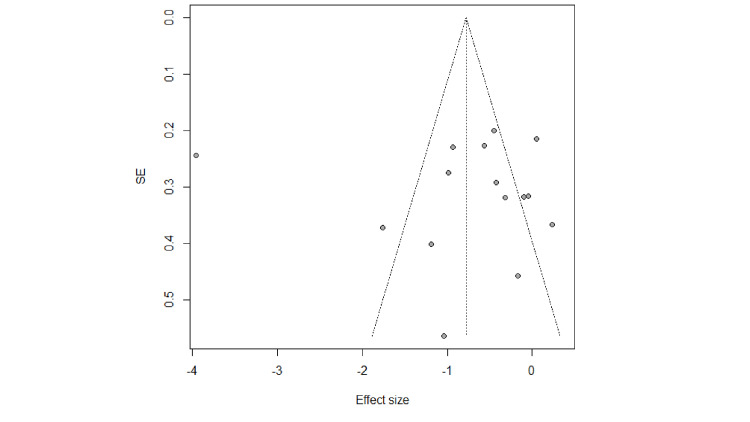
Funnel plot for SBP in cardiovascular patients with MBM intervention SBP- systolic blood pressure, MBM- mind-body medicine

**Figure 5:**
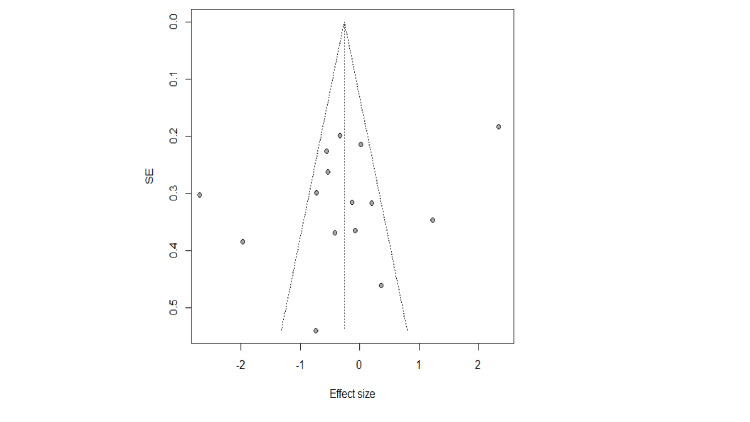
Funnel plot for DBP in cardiovascular patients with MBM intervention DBP- diastolic blood pressure, MBM- mind-body medicine

Findings of the Egger (P Egger=0.17, p=0.87) and a symmetry of the funnel plot (Figures 4, 5), suggest that there was no significant publication bias among studies. Sensitivity analysis results suggested that there was no essential change in the combined results or estimated heterogeneity after the exclusion of studies one by one.

Discussion

To the best of our knowledge, this is the first-ever meta-analysis on the effect of MBM intervention for blood pressure reduction in patients with cardiovascular diseases. Results of the present meta-analysis suggested reduction in systolic blood pressure with MBM interventions, such as meditation, mindfulness-based stress reduction and relaxation techniques among patients with cardiovascular disease, and a statistically insignificant difference for diastolic blood pressure. Subgroup analysis with the duration and frequency of MBM intervention also revealed the same for systolic blood pressure. The duration of the MBM interventions varied across the studies ranging from four weeks to 16 weeks. The majority of the included studies (n=7) used meditation as a MBM intervention followed by MBSR and breathing techniques.

Previous review done on MBM interventions on reduction in incidence of secondary cardiac events in patients with coronary heart disease concluded that MBM intervention can reduce the incidence of cardiac events (OR=0.33) and systolic blood pressure (Mean difference=-3.33 mmHg). However, the study stated that it does not reduce mortality (OR=0.8) and diastolic blood pressure (Mean difference= -1.30 mmHg) [9]. Findings from another meta-analysis also showed that MBM interventions were effective to combat stress, depression and blood pressure among patients with cardiac diseases [10]. They found moderate to low effects for the systolic (effect size: 0.48) and diastolic blood pressure reductions (effect size: 0.36) with MBM intervention. These findings were consistent with our current findings for systolic blood pressure and contradictory for diastolic blood pressure [10]. SBP increases the risk of secondary cardiac events and is also a predictor of cardiovascular illness. MBM can be an effective intervention to prevent secondary cardiac events, our meta-analysis is in line with previous studies.

To validate recent updates in the MBM research, we have included the studies published in the recent years, between 2000 and 2020 which might possibly be a limitation of the study as well. Another possible limitation is restricting to MBM interventions of meditation, mindfulness-based stress reduction and relaxation techniques, as few literature suggests other interventions such as taichi, qiqong and hypnotism as MBM interventions, which is beyond the scope of this review and are not included in this systematic review and meta-analysis. MBM technique is a lifestyle-based intervention and an emerging discipline, widely used in recent times [32]. However, the available scientific evidence on the beneficial effects of MBM is inconclusive. The current meta-analysis suggests MBM to patients with cardiovascular diseases, due to the clinically significant improvement in SBP.

Future implication or recommendation

Future studies on MBM and cardiovascular diseases should address specific research questions and future claims on the beneficial effects should be scientifically validated before accepting the same.

## Conclusions

The findings of the present meta-analysis do not provide substantial evidence to support the use of MBM in patients with cardiovascular diseases. While there was a clinically significant improvement observed in systolic blood pressure, the reduction in diastolic blood pressure was not statistically significant across the various MBM techniques. Due to the heterogeneous component of the intervention, more rigorous clinical trials have to be carried out in the future.
